# Replacement of anesthesia machines improves intraoperative ventilation parameters associated with the development of acute respiratory distress syndrome

**DOI:** 10.1186/1471-2253-14-44

**Published:** 2014-06-10

**Authors:** James M Blum, Victor Davila, Michael J Stentz, Ronald Dechert, Elizabeth Jewell, Milo Engoren

**Affiliations:** 1 Department of Anesthesiology, Emory University School of Medicine, 1364 Clifton Road NE, Atlanta, GA 30322, USA; 2 Emory Critical Care Center, Woodruff Health Sciences Center Administration Building, 1440 Clifton Road NE, Suite 313, Atlanta, GA 30322, USA; 3 Department of Anesthesiology, The Ohio State University, 410 W 10th Avenue, Columbus, OH 43210, USA; 4 Department of Anesthesiology, Division of Critical Care, The University of Michigan Health System, 1500 East Medical Center Drive, Ann Arbor, MI 48109-5861, USA; 5 Department of Respiratory Therapy, The University of Michigan Health Systems, 1500 East Medical Center Drive, Ann Arbor, MI 48109-5861, USA

**Keywords:** ARDS, Perioperative ventilation, Equipment upgrade, Study design

## Abstract

**Background:**

The impact of anesthetic equipment on clinical practice parameters associated with development of acute respiratory distress syndrome (ARDS) has not been extensively studied. We hypothesized a change in anesthesia machines would be associated with parameters associated with lower rates of ARDS.

**Methods:**

We performed a retrospective cohort study on a subset of data used to evaluate intraoperative ventilation. Patients included adults receiving a non-cardiac, non-thoracic, non-transplant, non-trauma, general anesthetic between 2/1/05, and 3/31/09 at the University of Michigan. Existing anesthesia machines (Narkomed IIb, Drager) were exchanged for new equipment (Aisys, General Electric). The initial subset compared the characteristics of patients anesthetized between 12/1/06 and 1/31/07 (pre) with those between 4/1/07 and 5/30/07 (post). An extended subset examined cases two years pre and post exchange. Using the standard predicted body weight (PBW), we calculated and compared the tidal volume (total Vt and mL/kg PBW) as well as positive end-expiratory pressure (PEEP), peak inspiratory pressure (PIP), Delta P (PIP-PEEP), and FiO2.

**Results:**

A total of 1,414 patients were included in the 2-month pre group and 1,635 patients included in the post group. Comparison of ventilation characteristics found statistically significant differences in median (pre v post): PIP (26 ± 6 v 21 ± 6 cmH2O,p < .001), Delta P (24 ± 6 v 19 ± 6 cmH2O, p < .001), Vt (588 ± 139 v 562 ± 121 ml, p < 0.001; 9.3 ± 2.2 v 9.0 ± 1.9 ml/kg predicted body weight, p < .001), FiO2 (0.57 ± 0.17 v 0.52 ± 0.18, p < .001). Groups did not differ in age, ASA category, PBW, or BMI. The two year subgroup had similar parameters. Risk adjustment resulted in minimal differences in the analysis. New anesthesia machines were associated with a non-statistically significant reduction in postoperative ARDS.

**Conclusions:**

In this study, a change in ventilator management was associated with an anesthesia machine exchange. The smaller Vt and lower PIP noted in the post group may imply a lower risk of volutrauma and barotrauma, which may be significant in at-risk populations. However, there was not a statistically significant reduction in the incidence of post-operative ARDS.

## Background

Low volume, low pressure mechanical ventilation has been shown to decrease mortality in critically ill patients with acute lung injury [1,2]. The intraoperative use of low tidal volumes has been associated with faster extubation and less reintubation in patients, while larger tidal volumes are associated with increased risk of postoperative organ dysfunction after cardiac surgery [3,4]. Larger tidal volumes are also a risk factor for respiratory failure after pneumonectomy [5]. Other clinical studies have shown that intraoperative low volume, low pressure ventilation decreases systemic inflammation and prevents pulmonary inflammation, and recent data suggests reduced drive pressure (∆P) may be associated with a reduction in post-operative acute respiratory distress syndrome (ARDS) [6-8]. Despite the putative benefits of intraoperative low volume, low pressure ventilation, particularly in patients at risk for ARDS, anesthesiologists rarely use low volume ventilation [4,9].

Until recently, the accurate delivery of preset tidal volumes was difficult. Anesthesiologists typically used mechanically-set volume-cycled ventilators to support patients intraoperatively. These ventilators allowed anesthesiologists to set the tidal volume and either the driving pressure or the inspiratory time (sometimes through manipulation of the respiratory rate and I:E ratio.) However, the actual volume delivered would vary based on breathing circuit compliance, chest compliance, and fresh gas flow. Newer microprocessor-controlled ventilators with automatic compensation for tube compliance and varying fresh gas flow have been shown to be more accurate at delivering small tidal volumes under conditions of both normal and low lung compliance [10]. They also allow the user more control over respiratory parameters.

We hypothesized that the change to a microprocessor-controlled ventilator would result in a lowering of intraoperative tidal volumes and lower inspiratory pressures associated with the development of ARDS.

## Methods

This study was a retrospective cohort analysis conducted using a subset of data previously described [8]. In brief, Institutional Review Board approval was obtained for this cohort study at The University of Michigan Health System (IRB-MED, Ann Arbor, MI), a large, quaternary care facility. All data were de-identified prior to analysis, and a waiver of consent was obtained for this study. All cases recorded in the anesthesia information management system (Centricity, General Electric Healthcare, Waukesha, WI) from 06/01/2004 to 06/01/2009 were screened for inclusion. Cases on the cardiac, thoracic, transplant, trauma, and vascular surgery services were excluded, as were cases with no recorded service.

Between February and March 2007, 63 anesthesia machines at the University of Michigan Hospital were exchanged in 8-10 room blocks from Drager Narkomed IIb to GE Aisys. Hence, all cases from February 1, 2007 to March 31, 2007 were excluded. No Narcomed devices were in use starting April 1, 2007. We then compared ventilation parameters on a subset for the two months before (December 1, 2006 to January 31, 2007) to the two months after (April 1, 2007 to May 30, 2007) to examine a possible immediate effect on ventilation. In order to determine if the change was durable and if there was an impact on development of ARDS, the two years before (February 1, 2005 to January 31, 2007) vs. the two years after (April 1, 2007 to March 31, 2009) the ventilator change were examined.

Preoperative data were prospectively collected from routine clinical documentation that was entered into the anesthesia information management system. The record includes a structured preoperative history and physical examination. Data abstracted from the preoperative history included basic demographic and comorbidity information necessary for the management of the critically ill or suggestive of the need for critical care services. A detailed description of variable definitions is included in previous publications [8]. Free text entries were hand-coded by the research team for analysis.

Unique hospital admissions were considered as the base unit for analysis. Admissions containing multiple anesthetic cases were analyzed from the last case of the admission or the last case prior to development of ARDS, as appropriate. Records from the final anesthetic of each admission were also used to determine preoperative comorbidities and ASA status. The ASA status recorded for this final case was collapsed into a binary variable reflecting whether a patient was considered ASA 1-2 or ASA 3-5.

Intraoperative physiologic and ventilator data were acquired using an automated, validated electronic interface from the anesthesia machines and physiologic monitors (Solar 9500; General Electric Healthcare). Fraction inspired oxygen (FiO2), peak inspiratory pressure (PIP), exhaled Vt, PEEP, oxyhemoglobin saturation (SpO2), drive pressure (∆P), and respiratory rate were obtained and analyzed for median values to eliminate spurious and isolated values. The number of ten-minute epochs of median PIP >30 cmH2O, and number of ten-minute epochs of median Vt > 12 cc/kg PBW from the time of incision to the end of anesthesia were examined as markers of continued high pressure and/or high volume ventilation.

Case times were validated using electronically documented heart rate from electrocardiogram or electronically documented start and end. Only cases with positive times were included. Cases from patients graded as ASA classification 6 were excluded.

When available, arterial blood gases that were manually entered by the anesthetic team into the anesthesia information management system were examined. From the recorded intraoperative PaO2 values and FiO2 the P/F ratio was calculated for each available blood gas. Volumes of crystalloid, colloid, units of packed erythrocytes (PRBC), units of fresh frozen plasma, and units of platelets were also obtained from the electronic anesthetic record.

The subpopulation of patients who went on to develop ARDS was identified from a prospectively collected research dataset of all adult critical care patients on ventilators at the University of Michigan Medical Center who were screened for entry into ARDS studies. Only patients receiving mechanical ventilation after their anesthetic were screened for ARDS. ARDS was diagnosed through analysis of the patient’s ventilator status, arterial blood gases, chest x-ray, and clinical documentation. Patients were deemed positive for the primary outcome of ARDS if they met the Berlin criteria for ARDS between postoperative days 0 and 7, inclusive. Charts of patients who developed ARDS on the day of their operation were examined by one of the authors (JMB), and those with a diagnosis of ARDS prior to their anesthetic were excluded. Finally, mortality data were collected from an institutional death database to compare the mortality of the risk-matched groups both with and without ARDS in order to determine the risk presented to patients who develop ARDS. This database is constructed using multiple resources including in-hospital mortality, failed follow-up at clinic visits and the social security death master file.

### Statistical Analysis

Statistical analysis was performed using R version 2.15.2 (R Foundation for Statistical Computing, Vienna, Austria). Descriptive statistics were used to summarize the before and after ventilator change groups. Differences between groups were tested using the chi-square test, Fisher’s exact test, t-test, or Mann-Whitney U test, as appropriate, with p-values < 0.05 indicating statistical significance.

To reduce confounding and determine if changes in population characteristics may have impacted the development of postoperative ARDS, we performed matching on the likelihood for developing postoperative ARDS within both the two-month and two-year cohorts. These scores were calculated using logistic regression on variables that likely increase the risk of postoperative ARDS, from our prior work [8]. This included preoperative patient characteristics as well as intraoperative use of blood products. Before-ventilator-change cases were then matched 1:1 with after-ventilator-change cases using a nearest neighbor match, with a caliper of .001 on the risk score. Standardized mean differences were calculated to assess balance in the variables included in the risk score, with values less than 10% indicating good balance. Appropriate descriptive statistics and tests were again used to compare variables between groups after matching.

## Results

**Figure 1:**
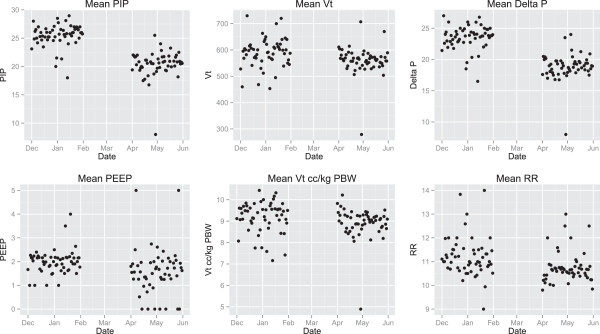
**Scatterplots of average ventilation parameters over 2 months before and 2 months after new machines are integrated into practice.** Significant changes were seen in peak inspiratory pressure (PIP) and drive pressure (∆P). Vt = tidal Volume, PEEP = positive end expiratory pressure, RR = respiratory rate.

A total of 1414 and 1635 cases were identified in the two-month periods before and after the anesthesia machine exchange. There were no statistically significant differences between the groups in their preoperative demographics (Table 1). Differences were found in the intraoperative use of colloids, the amount of crystalloid administered, and multiple ventilatory parameters. These included a substantial reduction in the peak inspiratory pressure, a decrease in the amount of PEEP, a decrease in the median Vt, and a decrease in the median drive pressure (Figure 1). There was also a substantial reduction in the number of epochs of high peak pressures (>30 cm H2O), and epochs of Vt greater than 12 cc/kg PBW, and an increase in the number of cases being managed with Vt of < 8 cc/kg PBW.

**Table 1:** Preoperative demographics and intraoperative data of patients 2 months before and after ventilator exchange

**Preoperative variable**	**Before vent exchange (n = 1414)**	**After vent exchange (n = 1635)**	**p**
**Age (mean ± SD, yr)**	51 ± 16	51 ± 16	0.713
**Male (n,%)**	628 (44%)	731 (44%)	0.898
**Patient ASA 3,4 or 5 (n,%)**	443 (31%)	536 (32%)	0.413
**Emergent Case (n,%)**	46 (3%)	54 (3%)	0.980
**Predicted Body Weight (mean ± SD, kg)**	64 ± 11	63 ± 10	0.316
**Weight (mean ± SD, kg)**	85 ± 24	85 ± 24	1
**BMI (mean ± SD, kg/m**^**2**^**)**	29 ± 7	29 ± 8	0.517
**Smoker**			
** Former (n,%)**	247 (18%)	304 (19%)	0.448
** Current (n,%)**	228 (16%)	267 (16%)	0.917
**Lung Disease (n,%)**	10 (0.7%)	8 (0.5%)	0.585
**Asthma (n,%)**	114 (8%)	134 (8%)	0.946
**COPD (n,%)**	73 (5%)	100 (6%)	0.291
**Diabetes (n,%)**	178 (13%)	218 (13%)	0.578
**Hypertension (n,%)**	506 (36%)	616 (38%)	0.297
**Coronary Artery Disease (n,%)**	93 (7%)	126 (8%)	0.257
**Congestive Heart Failure (n,%)**	40 (3%)	53 (3%)	0.579
**Chronic Kidney Disease (n,%)**	76 (5%)	110 (7%)	0.139
**Hepatic Disease (n,%)**	19 (1%)	33 (2%)	0.195
**Alcohol Abuse (n,%)**	45 (3%)	47 (3%)	0.697
**Current Steroid Therapy (n,%)**	46 (3%)	61 (4%)	0.538
**Intraoperative variable**	**Before vent exchange (n = 15,481)**	**After vent exchange (n = 18,945)**	**p**
**Ventilatory Parameters (case medians)**			
** PIP (mean ± SD, cmH**_**2**_**O)**	26 ± 6	21 ± 6	< 0.001
** PEEP (mean ± SD, cmH**_**2**_**O)**	2 ± 1.4	1.6 ± 2.6	< 0.001
Δ**P (mean ± SD, cmH**_**2**_**O)**	24 ± 6	19 ± 6	< 0.001
** RR (mean ± SD, breath per minute)**	11 ± 2.5	11 ± 3	< 0.001
** Vt (mean ± SD, ml)**	588 ± 139	562 ± 121	< 0.001
** Vt cc/kg PBW (mean ± SD, ml)**	9.3 ± 2.19	9.0 ± 1.88	< 0.001
** FiO**_**2 **_**(mean ± SD,%)**	57.2 ± 0.17	51.8 ± 18	< 0.001
** cc/kg PBW ≤ 6 (n,%)**	90 (6%)	104 (6%)	1
** cc/kg PBW ≤ 8 (n,%)**	316 (22%)	433 (27%)	0.009
**Epochs of 10 minutes:**			
** PIP > 30 cmH2O**	3.5 ± 7.5	1.5 ± 5	< 0.001
** Vt > 12 cc/kg PBW**	1.8 ± 5	0.9 ± 4	< 0.001
**Colloid use (n,%)**	198 (14%)	184 (11%)	0.026
**Crystalloid volume (median, 25**^**th**^**,75**^**th**^**, ml)**	2000 (1200,3000)	1900 (1000,2700)	< 0.001
**Case Length (mean ± SD, minutes)**	220 ± 124	208 ± 124	0.005
**Transfusion (n,%)**	79 (6%)	76 (5%)	0.248
**PRBC (n,%)**	77 (5%)	73 (5%)	0.244
**FFP (n,%)**	14 (1%)	17 (1%)	1
**Platelets (n,%)**	7(0.5%)	8 (0.5%)	0.813

ASA = American Society of Anesthesiology Classification, BMI = body mass index, COPD = chronic obstructive pulmonary disease, PIP = peak inspiratory pressure, PEEP = positive end expiratory pressure, RR = respiratory rate, Vt = tidal volume, PBW = predicted body weight, FiO2 = fraction inspired oxygen, PBW = predicted body weight, PRBC = packed red blood cells, FFP = fresh frozen plasma.

**Figure 2:**
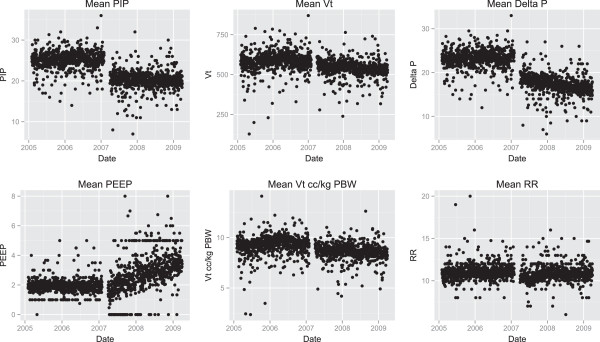
**Scatterplots of average ventilation parameters over 2 months before and 2 months after new machines are integrated into practice.** Significant changes were seen in peak inspiratory pressure (PIP) and drive pressure (∆P). Vt = Tidal Volume, PEEP = positive end expiratory pressure, RR = respiratory rate.

For the two-year pre/post cohorts, a total of 15,481 and 18,945 cases were identified before and after the anesthesia machine exchange. There were a number of statistically significant differences between the groups in their preoperative demographics (Table 2). In particular there was an increase in the preoperative use of steroids and an increase in patients who were former smokers. Intraoperative differences were extensive. The reductions in the ventilatory parameters over the two-month analysis were durable in the two-year analysis (Figure 2). In addition, there was a statistically significant reduction in use of blood products. A statistically significant increase in the use of colloids was not seen in the two-year cohort analysis.

**Table 2:** Preoperative demographics and intraoperative data of patients 2 years before and after ventilator exchange

**Preoperative variable**	**Before vent exchange (n = 15,481)**	**After vent exchange (n = 18,945)**	**p**
**Age (mean ± SD, yr)**	50 ± 17	52 ± 16	< 0.001
**Male (n,%)**	6936 (45%)	8619 (46%)	0.776
**Patient ASA 3,4 or 5 (n,%)**	4207 (27%)	6259 (33%)	< 0.001
**Emergent Case (n,%)**	697 (4%)	830 (4%)	0.605
**Predicted Body Weight (mean ± SD, kg)**	64 ± 11	64 ± 11	0.939
**Weight (mean ± SD, kg)**	83 ± 22	84 ± 22	< 0.001
**BMI (mean ± SD, kg/m**^**2**^**)**	28.6 ± 6.9	29.1 ± 7.3	< 0.001
**Smoker**			
** Former (n,%)**	2717 (18%)	4009 (21%)	< 0.001
** Current (n,%)**	2556 (17%)	3024 (16%)	0.174
**Lung Disease (n,%)**	45 (0.3%)	59 (0.3%)	0.802
**Asthma (n,%)**	1153 (7%)	1633 (9%)	< 0.001
**COPD (n,%)**	744 (5%)	1046 (6%)	0.003
**Diabetes (n,%)**	1758 (11%)	2658 (14%)	< 0.001
**Hypertension (n,%)**	5055 (33%)	7409 (39%)	< 0.001
**Coronary Artery Disease (n,%)**	1143 (7%)	1633 (9%)	< 0.001
**Congestive Heart Failure (n,%)**	378 (2%)	676 (4%)	0.545
**Chronic Kidney Disease (n,%)**	717 (5%)	1157 (6%)	< 0.001
**Hepatic Disease (n,%)**	235 (2%)	452 (2%)	< 0.001
**Alcohol Abuse (n,%)**	535 (4%)	582 (3%)	0.049
**Current Steroid Therapy (n,%)**	341 (2%)	658 (4%)	< 0.001
**Intraoperative variable**	**Before vent exchange (n = 15,481)**	**After vent exchange (n = 18,945)**	**p**
**Ventilatory Parameters (case medians)**			
** PIP (mean ± SD, cmH**_**2**_**O)**	25.4 ± 5.9	20.2 ± 6.1	< 0.001
** PEEP (mean ± SD, cmH**_**2**_**O)**	1.9 ± 1.2	2.7 ± 2.8	< 0.001
Δ**P (mean ± SD, cmH**_**2**_**O)**	23.4 ± 5.7	17.5 ± 5.6	< 0.001
** RR (mean ± SD, breath per minute)**	11.0 ± 2.5	10.7 ± 2.5	< 0.001
** Vt (mean ± SD, ml)**	587 ± 146	543 ± 118	< 0.001
** Vt cc/kg PBW (mean ± SD, ml)**	9.35 ± 2.29	8.67 ± 1.95	< 0.001
** FiO**_**2 **_**(mean ± SD,%)**	55.3 ± 16	54.1 ± 19	< 0.001
** cc/kg PBW ≤ 6 (n,%)**	1221 (8%)	1643 (9%)	< 0.001
** cc/kg PBW ≤ 8 (n,%)**	3534 (23%)	6465 (34%)	< 0.001
**Epochs of 10 minutes:**			
** PIP > 30 cmH2O**	3.1 ± 7.1	1.2 ± 4.2	< 0.001
** Vt > 12 cc/kg PBW**	1.92 ± 4.9	0.8 ± 3.4	< 0.001
**Colloid use (n,%)**	1585 (10%)	1828 (10%)	0.072
**Crystalloid volume (median, 25**^**th**^**,75**^**th**^**, ml)**	2000 (1200,3000)	2200 (1600,3100)	< 0.001
**Case Length (mean ± SD, minutes)**	214 ± 125	206 ± 122	< 0.001
**Transfusion (n,%)**	793 (5%)	880 (5%)	0.043
**PRBC (n,%)**	727 (5%)	810 (4%)	0.064
**FFP (n,%)**	175 (1%)	159 (1%)	0.007
**Platelets (n,%)**	91 (0.6%)	104 (0.5%)	0.685

ASA = American Society of Anesthesiology Classification, BMI = body mass index, COPD = chronic obstructive pulmonary disease, PIP = peak inspiratory pressure, PEEP = positive end expiratory pressure, RR = respiratory rate, Vt = tidal volume, PBW = predicted body weight, FiO2 = fraction inspired oxygen, PBW = predicted body weight, PRBC = packed red blood cells, FFP = fresh frozen plasma.

The risk score match resulted in 864 and 13250 matched pairs for two months and two years respectively. After matching, all standardized mean differences were less than 10%, indicating good balance between groups. No significant differences remained between the preoperative characteristics in the two matched groups (Tables 3 and 4). After matching on preoperative risk, there remained a statistically significant reduction in many parameters associated with the development of ARDS and a statistically non-significant reduction in the incidence of postoperative ARDS (Tables 5 and 6). There was no reduction in mortality in any group.

**Table 3:** Preoperative demographics and intraoperative data of patients 2 months before and after ventilator exchange after risk matching

**Preoperative variable**	**Before vent exchange (n = 864)**	**After vent exchange (n = 864)**	**p**
**Age (mean ± SD, yr)**	49 ± 16	48 ± 16	0.656
**Male (n,%)**	358 (41%)	362 (42%)	0.884
**Patient ASA 3,4 or 5 (n,%)**	198 (23%)	188 (22%)	0.603
**Emergent Case (n,%)**	24 (3%)	16 (2%)	0.263
**Predicted Body Weight (mean ± SD, kg)**	64 ± 10.5	63 ± 10.5	0.294
**Weight (mean ± SD, kg)**	84 ± 23.5	84 ± 22.8	0.658
**BMI (mean ± SD, kg/m**^**2**^**)**	28.9 ± 7.4	29.0 ± 7.3	0.854
**Smoker**			
** Former (n,%)**	141 (16%)	134 (16%)	0.693
** Current (n,%)**	138 (16%)	132 (15%)	0.74
**Lung Disease (n,%)**	3 (0.3%)	5 (0.6%)	0.726
**Asthma (n,%)**	71 (8%)	72 (8%)	1
**COPD (n,%)**	20 (2%)	19 (2%)	1
**Diabetes (n,%)**	86 (10%)	73 (8%)	0.381
**Hypertension (n,%)**	250 (29%)	230 (27%)	0.308
**Coronary Artery Disease (n,%)**	23 (3%)	18 (2%)	.527
**Congestive Heart Failure (n,%)**	12 (1%)	12 (1%)	1
**Chronic Kidney Disease (n,%)**	21 (2%)	20 (2%)	1
**Hepatic Disease (n,%)**	11 (1%)	11 (1%)	1
**Alcohol Abuse (n,%)**	27 (3%)	21 (2%)	0.464
**Current Steroid Therapy (n,%)**	24 (3%)	25 (3%)	1
**Intraoperative variable**	**Before vent exchange (n = 864)**	**After vent exchange (n = 864)**	**p**
**Ventilatory Parameters (case medians)**			
** PIP (mean ± SD, cmH**_**2**_**O)**	25.3 ± 5.7	20.3 ± 6.2	< 0.001
** PEEP (mean ± SD, cmH**_**2**_**O)**	2.0 ± 1.3	1.5 ± 2.5	< 0.001
Δ**P (mean ± SD, cmH**_**2**_**O)**	23.3 ± 5.4	18.8 ± 5.6	< 0.001
** RR (mean ± SD, breath per minute)**	11.0 ± 2.3	10.5 ± 2.2	< 0.001
** Vt (mean ± SD, ml)**	587 ± 146	559 ± 125	< 0.001
** Vt cc/kg PBW (mean ± SD, ml)**	9.33 ± 2.22	8.98 ± 1.94	< 0.001
** FiO**_**2 **_**(mean ± SD,%)**	55.5 ± 16	51.2 ± 18	< 0.001
** cc/kg PBW ≤ 6 (n,%)**	62 (7%)	63 (7%)	1
** cc/kg PBW ≤ 8 (n,%)**	188 (22%)	219 (34%)	0.089
** Epochs of 10 minutes:**			
** PIP > 30 cmH2O**	2.9 ± 6.4	1.4 ± 4.5	< 0.001
** Vt > 12 cc/kg PBW**	1.7 ± 4.3	0.9 ± 3.6	< 0.001
**Colloid use (n,%)**	51 (5.9%)	47 (5.4%)	0.755
**Crystalloid volume (median, 25**^**th**^**,75**^**th**^**, ml)**	1950 (1100, 2700)	1900 (1000,2500)	0.111
**Case Length (mean ± SD, minutes)**	198 ± 103	195 ± 104	0.531
**Transfusion (n,%)**	17 (2%)	15 (2%)	0.858
**PRBC (n,%)**	16 (2%)	15 (5%)	1
**FFP (n,%)**	2 (0.2%)	4 (0.5%)	0.683
**Platelets (n,%)**	2 (0.2%)	1 (0.1%)	1

ASA = American Society of Anesthesiology Classification, BMI = body mass index, COPD = chronic obstructive pulmonary disease, PIP = peak inspiratory pressure, PEEP = positive end expiratory pressure, RR = respiratory rate, Vt = tidal volume, PBW = predicted body weight, FiO2 = fraction inspired oxygen, PBW = predicted body weight, PRBC = packed red blood cells, FFP = fresh frozen plasma.

**Table 4:** Preoperative demographics and intraoperative data of patients 2 years before and after ventilator exchange after risk matching

**Preoperative variable**	**Before vent exchange (n = 13,250)**	**After vent exchange (n = 13,250)**	**p**
**Age (mean ± SD, yr)**	51 ± 16	50 ± 16	< 0.001
**Male (n,%)**	5932 (45%)	5956 (45%)	0.776
**Patient ASA 3,4 or 5 (n,%)**	3788 (28.6%)	3604 (27.2%)	0.012
**Emergent Case (n,%)**	591 (4.5%)	588 (4.4%)	0.952
**Predicted Body Weight (mean ± SD, kg)**	63 ± 10.6	64 ± 10.6	0.247
**Weight (mean ± SD, kg)**	83 ± 22	83 ± 22	0.568
**BMI (mean ± SD, kg/m**^**2**^**)**	29 ± 7	29 ± 7	0.906
**Smoker**			
** Former (n,%)**	2410 (18.2%)	2637 (19.9%)	< 0.001
** Current (n,%)**	2182 (16.5%)	2195 (16.6%)	0.843
**Lung Disease (n,%)**	37 (0.3%)	33 (0.2%)	0.72
**Asthma (n,%)**	1054 (8%)	998 (7.5%)	0.206
**COPD (n,%)**	664 (5%)	643 (4.9%)	0.57
**Diabetes (n,%)**	1531 (12%)	1477 (11%)	0.305
**Hypertension (n,%)**	4670 (35%)	4366 (33%)	< 0.001
**Coronary Artery Disease (n,%)**	1028 (8%)	989 (7%)	0.379
**Congestive Heart Failure (n,%)**	307 (2%)	323 (2%)	0.545
**Chronic Kidney Disease (n,%)**	616 (5%)	605 (5%)	0.77
**Hepatic Disease (n,%)**	212 (2%)	277 (2%)	0.003
**Alcohol Abuse (n,%)**	467 (4%)	396 (3%)	0.015
**Current Steroid Therapy (n,%)**	304 (2%)	415 (3%)	< 0.001
**Intraoperative Variable**	**Before vent exchange (n = 13,250)**	**After vent exchange (n = 13,250)**	**p**
**Ventilatory Parameters (case medians)**			
** PIP (mean ± SD, cmH**_**2**_**O)**	25.5 ± 6	20 ± 6	< 0.001
** PEEP (mean ± SD, cmH**_**2**_**O)**	1.9 ± 1.2	2.7 ± 2.8	0.257
Δ**P (mean ± SD, cmH**_**2**_**O)**	23.6 ± 6	17.3 ± 6	< 0.001
** RR (mean ± SD, breath per minute)**	11 ± 2.5	11 ± 2.5	< 0.001
** Vt (mean ± SD, ml)**	590 ± 144	541 ± 120	< 0.001
** Vt cc/kg PBW (mean ± SD, ml)**	9.41 ± 2.26	8.62 ± 1.96	< 0.001
** FiO**_**2 **_**(mean ± SD)**	55 ± 16	54 ± 19	< 0.001
** cc/kg PBW ≤ 6 (n,%)**	953 (7%)	1223 (9%)	< 0.001
** cc/kg PBW ≤ 8 (n,%)**	2915 (22%)	4584 (35%)	< 0.001
**Epochs of 10 minutes:**			
** PIP > 30 cmH2O**	3.2 ± 7.1	1.1 ± 4	< 0.001
** Vt > 12 cc/kg PBW**	2 ± 4.8	0.7 ± 3.1	< 0.001
**Colloid use (n,%)**	1202 (9%)	1157 (9%)	0.343
**Crystalloid volume (median, 25**^**th**^**,75**^**th**^**, ml)**	2000 (1300,3000)	2000 (1500,3000)	< 0.001
**Case Length (mean ± SD, minutes)**	215 ± 122	197 ± 116	< 0.001
**Transfusion (n,%)**	560 (4%)	554 (4%)	0.878
**PRBC (n,%)**	509 (4%)	513 (4%)	0.924
**FFP (n,%)**	103 (0.8%)	91 (0.7%)	0.428
**Platelets (n,%)**	64 (<0.5%)	60 (<0.5%)	0.787

ASA = American Society of Anesthesiology Classification, BMI = body mass index, COPD = chronic obstructive pulmonary disease, PIP = peak inspiratory pressure, PEEP = positive end expiratory pressure, RR = respiratory rate, Vt = tidal volume, PBW = predicted body weight, FiO2 = fraction inspired oxygen, PBW = predicted body weight, PRBC = packed red blood cells, FFP = fresh frozen plasma.

**Table 5:** 28 day and 90 day mortality and acute lung injury incidence after matching on the 2 month cohort

**Variable**	**Before vent exchange (n = 864)**	**After vent exchange (n = 864)**	**p**
**28 day Mortality**	2 (0.2%)	3 (0.3%)	1
**90 day Mortality**	8 (0.9%)	6 (0.7%)	0.79
**Acute Lung Injury within 7 days**	3 (0.3%)	0 (0.0%)	0.25

**Table 6:** 28 day and 90 day mortality and acute lung injury incidence after matching on the 2 year cohort

**Variable**	**Before vent exchange (n = 13,250)**	**After vent exchange (n = 13,250)**	**p**
**28 day Mortality**	83 (0.6%)	74 (0.6%)	0.522
**90 day Mortality**	190 (1.4%)	188 (1.4%)	0.959
**Acute Lung Injury within 7 days**	20 (0.2%)	12 (0.1%)	0.216

## Discussion

The conclusions of this study are 1) The introduction of a new anesthesia machine with advanced ventilator capabilities was associated with immediate and dramatic reduction in the PIP delivered to patients, 2) There was also a statically significant reduction in the Vt delivered to patients, the number of epochs greater than 12 cc/kg PBW, and epochs where patients received peak inspiratory pressures greater than 30 cm H2O, 3) The reductions in these settings were durable over a two-year period, 4) The settings were consistent with settings that have been associated with a reduction in the incidence of ARDS, and 5) There was a non-significant reduction in the incidence of ARDS after the introduction of the new anesthesia machine. Despite a significant change in the patient population, this non-significant reduction remained after matching.

In our previous work, we demonstrated that the development of postoperative ARDS was associated with a high mortality rate. We also found that intraoperative ventilator management, in particular the reduction of the ∆P, may lessen the risk of developing ARDS [8].

Our institution’s introduction of a new anesthesia machine that incorporated advanced microprocessor-controlled ventilation into all operating rooms nearly simultaneously offered a unique opportunity to examine the impact of ventilator features on ventilation parameters. We have noted a continued reduction in pressures and volumes over a several year period with a distinct reduction in tidal volumes and pressures seen from 2007 to 2008 [11].

This new analysis of data from our prior study offered the opportunity to determine if favorable changes in ventilation occurred with the introduction of advanced equipment. Furthermore, it offered the opportunity to observe if there was a reduction in the development of ARDS with the use of these improved parameters. While a statistically significant reduction in ARDS was not demonstrated in this study, there was a significant change in the ventilation parameters associated with the development of the condition.

The concept of ventilator induced lung injury has been well established, and its avoidance is the currently preferred prevention and treatment modality for ARDS [12]. Although the majority of published prospective research has focused on reduced tidal volumes as the primary endpoint, there exists substantial evidence that increased ventilator pressures may be highly predictive of ARDS associated mortality. In the ARDSnet ARMA trial, a target ventilation of 4-8 cc/kg PBW with plateau pressures < 30 cm H2O was compared to a ventilation strategy using volumes of 12 cc/kg PBW and plateau pressures < 50 cm H2O [2]. The absolute mortality reduction was found to be 9% in favor of the low tidal volume, low pressure group.

Additional data supports the reduction in pressures to avoid the development of ARDS, particularly in the perioperative period. Licker and colleagues described the hyperpressure index and its prediction of post-operative ARDS in the thoracic surgery population [13,14]. In our prior work, positive predictors of the development of ARDS included higher ∆P and higher FiO2 [8].

New ventilator technologies have been demonstrated to reduce the PIP during ventilation and by definition produce lower ∆P. The premise behind these reduced pressures is the use of a decelerating flow profile based on pressure. Functionally, this was been demonstrated by Rappaport and colleagues in a trial of volume controlled ventilation vs. pressure limited ventilation in critically ill patients suffering from respiratory failure [15]. The study demonstrated lower PIP in addition to accelerated improvements in lung compliance. Guldager et al. also demonstrated a reduction in PIP using pressure control with a volume guarantee in patients with acute respiratory failure using tidal volumes from 5 to 8 cc/kg PBW [16]. Despite the previous literature from the critical care setting, this is the first evidence, to our knowledge, demonstrating a change in ventilation parameters with the introduction of a new anesthesia machine. The changes in parameters were consistent with strategies suggested to reduce the incidence of ARDS. While the data did not demonstrate this reduction we believe this may very well be due to inadequate power. Nearly 30,000 patients would have to be enrolled in each group to achieve a statistical power of 90%. Many of the ventilator changes could be the result of a change in patient population and changes in anesthetic technique, including a reduction in transfusions. To address these concerns, we created a risk-matched cohort to examine differences within the groups. While there continued to be a reduction in ARDS cases, the reduction remained statistically insignificant.

This study has several limitations. Data were collected as part of routine clinical care and were not subject to a validation processes typically used in prospective trials. Although data were entered using a predefined selection process for each variable, there was no formal training on the definitions for each variable. Free text, which is allowed in all fields, was left to interpretation by the research team. Despite these data collection limitations, such data have been used frequently to develop models in the literature and have correlated with data that were prospectively collected by dedicated research staff [17-19]. Additionally, the method of data collection regarding tidal volumes differed between the two anesthesia machines and was not able to be corrected for. The Aisys machine uses a corrected tidal volume accounting for volumes lost due to circuit compliance whereas the Narkomed IIb utilized a simple spirometer that did not provide any correction. It is conceivable this may impact the total Vt to such an extent that any documented change may indeed be non-significant, as the compliance for a typical adult circuit on the Narkomed IIb has been documented to be 2.75 ml/cm H2O. However, this should not have impacted the other observed ventilation changes. Other reasons for the potential change include the mode of ventilation. The Narkomed IIb only provided one mode of mechanical ventilation, volume controlled intermittent mandatory ventilation with a maximal pressure setting that was manually set. The Aisys provides a multitude of ventilation settings including various forms of volume controlled and pressure controlled ventilation. Unfortunately, the mode of ventilation is not recorded in our AIMS, but the standard setting is pressure control with a volume guarantee, which, from our experience, is by far the most common mode used in our patient population. Furthermore, plateau pressures are not recorded. Instead, we used PIP and ΔP as surrogates. Next, the data are from a single, large, tertiary care center, collected over several years. While this is required for such an analysis, the patient population may have changed with the introduction of the new anesthesia machines. The database used to determine whether patients had ARDS was developed using a screening mechanism requiring mechanical ventilation. Thus, it is possible the true frequency of ARDS is underrepresented. Additionally, we cannot be completely confident the changes in ventilation parameters were only due to the anesthesia machines and not due to a natural change in ventilation decisions by the anesthesia providers. However, we believe that changes during the two month periods before and after machine exchange would be unlikely due to natural changes. Finally, the mortality data are based on an internal death registry and may not capture mortality of patients who were discharged to another long-term facility for ongoing care.

## Conclusions

Despite the limitations, this investigation provides evidence that perioperative ventilation changes with the introduction of new anesthesia machine. This may provide a new mechanism for the study of intraoperative mechanical ventilation and its impact on the development of ARDS. We have demonstrated that a new anesthesia machine is clearly associated with a reduction in ventilation parameters that are correlated with the development of ARDS. The data were underpowered to demonstrate a reduction in ARDS but was associated with a non-statistically significant reduction in ARDS. Additional studies are required to determine if such effect is seen at other institutions.

## Abbreviations

ARDS: Acute respiratory distress syndrome; PBW: Predicted body weight; Vt: Tital volume; PEEP: Positive end-expiratory pressure; PIP: Peak inspiratory pressure; Delta P: Peak inspiratory pressure-positive end-expiratory pressure; FiO2: Fraction inspired oxygen; ASA: American Society of Anesthesiology classification; BMI: Body mass index; ∆P: Drive pressure; IRB-MED: Institutional Review Board at the University of Michigan Health System in Ann Arbor, MI; SpO2: Oxyhemoglobin saturation; PaO2: Partial pressure of oxygen in arterial blood; PRBC: Packed red blood cells; JMB: James Marlow Blum; R version 2.15.2: R Foundation for Statistical Computing, Vienna, Austria; ARDSnet: Acute respiratory distress syndrome network; AIMS: Anesthesia information system; RR: Respiratory rate; COPD: Chronic Obstructive Pulmonary Disease; FFP: Fresh frozen plasma.

## Competing interests

The authors declare that they have no competing interests.

## Authors’ contributions

JB collected data, analyzed the data, and assisted in manuscript composition. VD collected data, analyzed the data. MS analyzed the data. RD collected data, analyzed the data. EJ analyzed the data, assisted in manuscript composition. ME analyzed the data, assisted in manuscript composition. All authors read and approved the final manuscript.

## Pre-publication history

The pre-publication history for this paper can be accessed here:

http://www.biomedcentral.com/1471-2253/14/44/prepub
